# The Differential Expression of Core Genes in Nucleotide Excision Repair Pathway Indicates Colorectal Carcinogenesis and Prognosis

**DOI:** 10.1155/2018/9651320

**Published:** 2018-01-15

**Authors:** Jingwei Liu, Hao Li, Liping Sun, Xue Feng, Zhenning Wang, Yuan Yuan, Chengzhong Xing

**Affiliations:** Tumor Etiology and Screening Department, Cancer Institute and General Surgery, The First Hospital of China Medical University and Key Laboratory of Cancer Etiology and Prevention, Liaoning Provincial Education Department, China Medical University, Shenyang 110001, China

## Abstract

**Background:**

Nucleotide excision repair (NER) plays a critical role in maintaining genome integrity. This study aimed to investigate the expression of NER genes and their associations with colorectal cancer (CRC) development.

**Method:**

Expressions of NER genes in CRC and normal tissues were analysed by ONCOMINE. The Cancer Genome Atlas (TCGA) data were downloaded to explore relationship of NER expression with clinicopathological parameters and survival of CRC.

**Results:**

ERCC1, ERCC2, ERCC5, and DDB2 were upregulated while ERCC4 was downregulated in CRC. For colon cancer, high ERCC3 expression was related to better T stage; ERCC5 expression indicated deeper T stage and distant metastasis; DDB2 expression suggested earlier TNM stage. For rectal cancer, ERCC2 expression correlated with favourable T stage; XPA expression predicted worse TNM stage. ERCC2 expression was associated with worse overall survival (OS) in colon cancer (HR = 1.53, *P* = 0.043). Colon cancer patients with high ERCC4 expression showed favorable OS in males (HR = 0.54, *P* = 0.035). High XPC expression demonstrated decreased death hazards in rectal cancer (HR = 0.40, *P* = 0.026).

**Conclusion:**

ERCC1, ERCC2, ERCC4, ERCC5, and DDB2 were differently expressed in CRC and normal tissues; ERCC2, ERCC3, ERCC5, XPA, and DDB2 correlated with clinicopathological parameters of CRC, while ERCC2, ERCC4, and XPC might predict CRC prognosis.

## 1. Introduction

As one of the leading cause of cancer-related mortality worldwide, colorectal cancer (CRC) develops from normal epithelial cells through benign adenomas ultimately to malignant carcinomas [1]. Although several key genes such as* APC*,* TP53,* and* KRAS* have been identified to be implicated in the initiation and progression of CRC [2–4], robust biomarkers which could predict risk and clinical outcome of CRC are still required [5].

DNA damage resulted from endogenous and exogenous stimuli that can give rise to multiple biological disorders and diseases including cancer [6]. DNA repair system could repair harmful DNA damage, of which nucleotide excision repair (NER) could repair various DNA damage, including UV-induced cyclobutane pyrimidine dimers, DNA crosslinks, and bulky adducts [7]. The NER process consists of several key links including recognition, demarcation and unwinding, incision, and ligation of new strand [8]. Different key proteins are involved in their corresponding step: XPA, XPC, DDB1, DDB2, ERCC6 (CSB), and ERCC8 (CSA) are responsible for DNA damage recognition; ERCC2 (XPD) and ERCC3 (XPB) accomplish 5′-3′ and 3′-5′ unwinding of the DNA strands of the damaged site, while the damaged DNA is excised at 5′ site by XPF (ERCC4)-ERCC1 heterodimer and at 3′ site by ERCC5 (XPG) [9, 10]. Aberrant expression of key NER factors alters NER capacity, thus threatening genomic stability and integrity [11]. Unrepaired DNA damage would have deleterious effects to normal biological functions of cells and contribute to the development of CRC [12]. Therefore, the expression profiling of NER pathway members might imply great significance in colorectal carcinogenesis and progression.

So far, although a number of investigations have focused on the role of NER genes in CRC [13–16], no comprehensive study has evaluated the whole picture of entire NER family members from the perspective of expression characteristics and prognostic role in CRC. In order to elucidate the expression profile and prognostic role of core NER pathway members (ERCC1, ERCC2, ERCC3, ERCC4, ERCC5, ERCC6, ERCC8, XPA, XPC, DDB1, and DDB2) in CRC, we performed comprehensive analysis by using available datasets of ONCOMINE and TCGA (The Cancer Genome Atlas). The differential expression of key NER pathway members was analysed in CRC and normal intestinal tissues. In addition, the association of expression of the involved NER genes with clinicopathological parameters and prognosis of CRC was investigated.

## 2. Materials and Methods

### 2.1. ONCOMINE Database Analysis

ONCOMINE database is a public available microarray database (https://www.oncomine.org/) which discovers genes that are differently expressed in cancer and normal tissues [17]. ONCOMINE contains microarray information of more than 86000 samples from 715 datasets, which also offers online statistical analysis. Student's *t*-test was performed to compare the different expression of NER pathway members in cancer tissues and its corresponding normal tissues. The cut-off *p* value and fold change were defined as 0.01 and 2, respectively.

### 2.2. Obtainment of Data Form TCGA Database

The Cancer Genome Atlas (TCGA) is a public available database (https://cancergenome.nih.gov/) which is a collaboration between the National Cancer Institute (NCI) and the National Human Genome Research Institute (NHGRI) that has generated comprehensive, multidimensional maps of the important genomic changes in 33 types of cancer [18]. Over 11,000 patients with tumor tissue and matched normal tissues were included in TCGA dataset, whose genomic information bring great improvement to the prevention, diagnosis, and treatment of diverse types of cancer.

In this study, data of 478 colon adenocarcinoma cases (TCGA-COAD, provisional) with expression and clinicopathological information was downloaded for further analysis. Additionally, data of 166 rectum adenocarcinoma (TCGA-READ, provisional) was obtained to analyse the relationship of NER pathway members expression with clinical outcome.

### 2.3. Statistical Analysis of TCGA Data

R language (Version 3.4.1) was used to analyse the data obtained from TCGA. The median value of mRNA expression was adopted to differentiate high expression and low expression of certain NER factor. The *χ*^2^ test was applied to assess the relationship between NER member expression and clinicopathological parameters such as TNM stage and recurrence. We employed the Kaplan-Meier method to visualize overall survival (OS) differentiated by expression level. The log-rank test was performed to test for equality of the survival distributions. Crude or adjusted hazards ratios (HR) and 95% confidence intervals (CI) of each NER members were calculated through univariate and multivariate Cox proportional hazards models to estimate its effect on OS with or without adjustment for confounding factors. Variables including age, sex and TNM stage were further adjusted by multivariate Cox proportional hazards regression models to evaluate the independent prognostic value of NER members. Two-tailed *P* values < 0.05 were regarded as statistically significant.

## 3. Results

### 3.1. ERCC1, ERCC2, ERCC4, ERCC5, and DDB2 Are Differently Expressed in CRC and Normal Tissues

**Figure 1:**
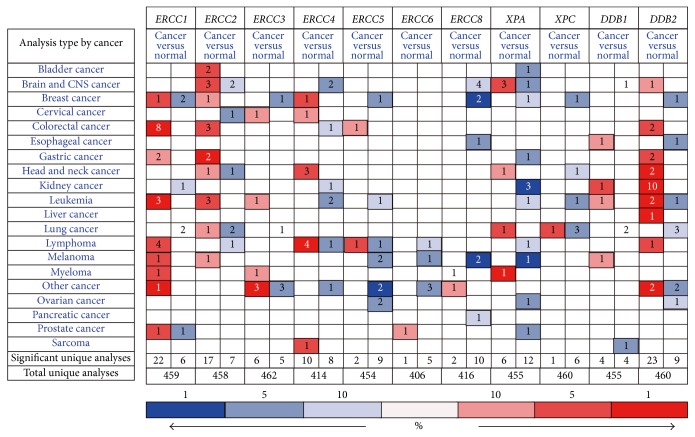
*The mRNA expression level of NER pathway genes in different types of cancers according to ONCOMINE database*. Red: upregulation; blue: downregulation.

**Figure 2:**
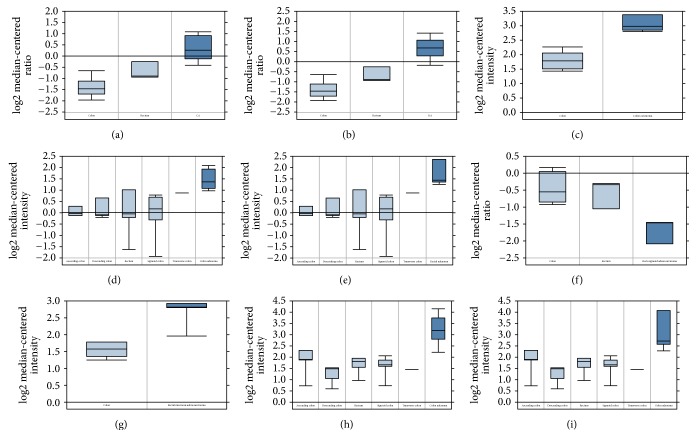
*Box plots that represent the mRNA expression level of NER pathway genes in different types of CRC*. (a) ERCC1: colon adenocarcinoma versus normal; (b) ERCC1: rectal adenocarcinoma versus normal; (c) ERCC1: colon carcinoma versus normal; (d) ERCC2: colon adenoma versus normal; (e) ERCC2: rectal adenoma versus normal; (f) ERCC4: rectosigmoid adenocarcinoma versus normal; (g) ERCC5: rectal mucinous adenocarcinoma versus normal; (h) DDB2: colon adenoma versus normal; (i) DDB2: rectal adenoma versus normal.

The detailed information of location and function for core NER pathway members was summarized in Table 1. According to the analysing results of ONCOMINE, Figure 1 suggested the expression differentiation of NER genes in all types of cancer and its matched normal tissues. ERCC1, ERCC2, ERCC5, and DDB2 were highly expressed in CRC tissues compared to matched normal tissues, while ERCC4 was found to be downregulated in CRC (Table 2). In both colon adenocarcinoma (fold change = 3.075, *P* = 1.67*E* − 13) and rectal adenocarcinoma (fold change = 3.813, *P* = 1.79*E* − 16), ERCC1 was consistently upregulated in cancer tissues. On the basis of Sabates-Bellver Colon dataset [19], overexpression of ERCC2 was detected in both colon adenoma and rectal adenoma, with fold change of 2.391 and 2.813, respectively. Another NER member with significantly increased mRNA expression was ERCC5 in rectal mucinous adenocarcinoma (fold change = 2.121, *P* = 0.005) according to Kaiser Colon dataset. Besides, colon adenoma and rectal adenoma both demonstrated upregulated mRNA of DDB2 (fold change is 3.159 and 2.890, resp.) in Sabates-Bellver Colon dataset. The only one downregulated NER member was ERCC4 in rectosigmoid adenocarcinoma with fold change of −2.271 (*P* = 0.009). The significant alternations of expression of NER members in specific subtype of CRC were visualized by box plot in Figure 2.

### 3.2. ERCC2, ERCC3, ERCC5, XPA, and DDB2 Correlated with Clinicopathological Parameters of CRC

Relationship between expression of NER members and clinicopathological parameters of colon cancer and rectal cancer was summarized in Supplementary Tables 1 and 2. Significant associations were shown in Table 3. For colon cancer, ERCC3 high expression was related with better T stage (*P* = 0.011); increased ERCC5 expression indicated deeper invasion of T stage (*P* = 0.040) and presence of distant metastasis (*P* = 0.015); DDB2 high expression suggested earlier TNM stage (*P* = 0.005) and absence of lymph node (*P* = 0.020) or distant metastasis (*P* = 0.012). For rectal cancer, significant relation was observed between ERCC2 high expression and favourable T stage (*P* = 0.019); high XPA expression obviously predicted worse TNM stage (*P* = 0.025), T stage (*P* = 0.019), and N stage (*P* = 0.008). In addition, ERCC5 and ERCC6 showed marginally significant association with recurrence of rectal cancer with a P value of 0.51 (Supplementary Table 2).

### 3.3. ERCC2, ERCC4, and XPC Associated with Prognosis of CRC

**Figure 3:**
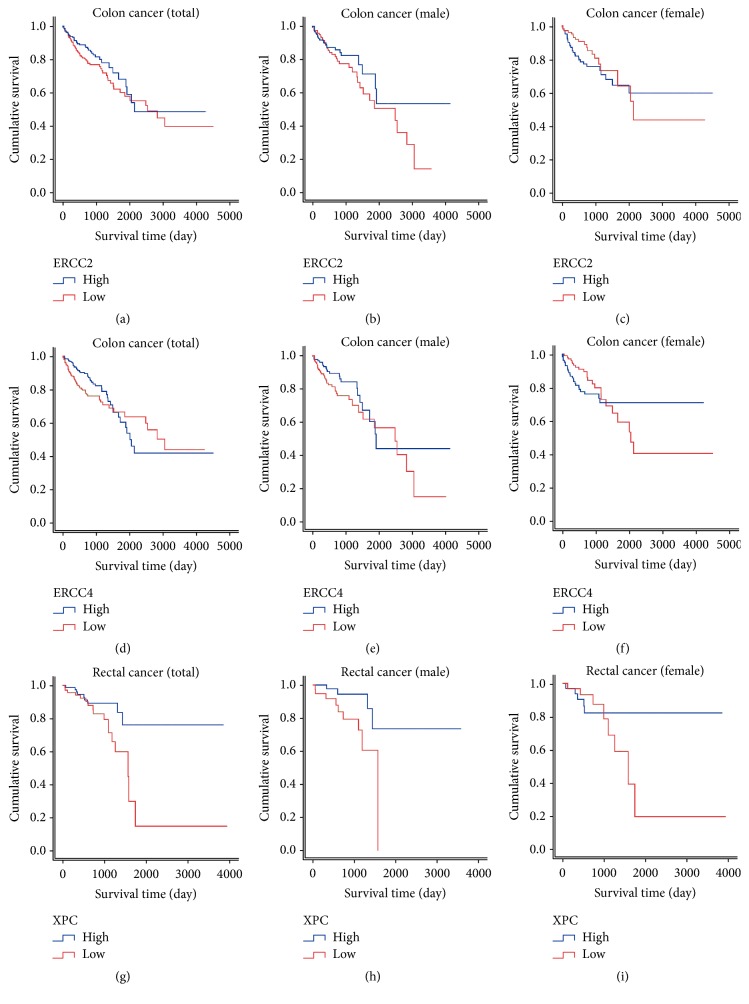
*Kaplan-Meier survival curves by the expression level of NER pathway members in CRC prognosis*. (a) ERCC2: total patients; (b) ERCC2: males; (c) ERCC2: females; (d) ERCC4: total patients; (e) ERCC4: males; (f) ERCC4: females; (g) XPC: total patients; (h) XPC: males; (i) XPC: females.

Role of NER members' expression in the prediction of CRC prognosis was summarized in Table 4. Multivariate analysis indicated that high ERCC2 expression was associated with worse overall survival (OS) of colon cancer (adjusted HR = 1.53, 95% CI = 1.01–2.31, *P* = 0.043). Subgroup based on gender suggested a more significant result in males (adjusted HR = 1.84, 95% CI = 1.04–3.26, *P* = 0.037) while none significant outcome was observed in females (adjusted HR = 1.22, 95% CI = 0.66–2.24, *P* = 0.532). Colon cancer patients with high expression of ERCC4 showed significantly favourable OS than those with low ERCC4 expression according to multivariate analysis in males (adjusted HR = 0.54, 95% CI = 0.30–0.96, *P* = 0.035). As for rectal cancer, subjects with high XPC expression demonstrated significantly decreased hazards of death in univariate model (HR = 0.40, 95% CI = 0.18–0.89, *P* = 0.026). Males patients with high XPC expression exhibited longer OS than those with low expression (HR = 0.26, 95% CI = 0.08–0.88, *P* = 0.030) while the results of females did not reach statistical significance. Kaplan-Meier plots which visualize overall survival differentiated by NER expression level were displayed in Figure 3.

## 4. Discussion

**Figure 4:**
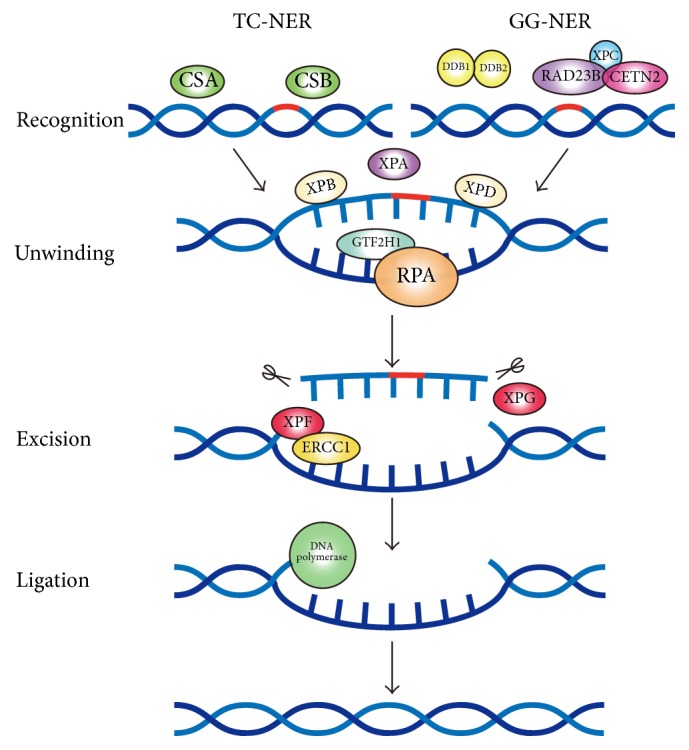
Schematic diagram of nucleotide excision repair (NER) pathway.

NER consists of transcription-coupled nucleotide excision repair (TCNER) and global genome nucleotide excision repair (GGNER) [20], each step of which requires specific NER members to accomplish functions including recognition, unwinding, and excision (Figure 4). Until now, a number of investigations have focused on the role of NER genes in CRC, but most studies were dispersed without an overview of the impact of core factors implicated in entire NER process on the development, progression, and prognosis of CRC. This investigation, for the first time, elaborated on expression profiling of whole members in NER pathway, which orchestrate a complex and critical aspect in CRC pathogenesis and clinical outcome. We finally elucidated that each procedure (recognition, unwinding, and excision) of NER pathway was indispensable for the successful repair, and the aberrant changes of key involved factors led to alternations of CRC progression and outcome.

Results from our study suggested that ERCC1, ERCC2, ERCC5, and DDB2 were highly expressed in CRC compared to matched normal tissues, while ERCC4 was found to be downregulated in CRC. ERCC1–ERCC4 heterodimer is responsible for the 5′ site excision while the incision of impaired DNA at the 3′ site is performed by ERCC5 [21, 22]. ERCC2 participates in 5′-3′ unwinding of the DNA strands of the damaged site [23]. DDB2 forms a complex with DDB1 to ensure successful GG-NER recognition [24]. The overexpression of ERCC1, ERCC2, ERCC5, and DDB2 in CRC might arise from the accumulation of abnormally damaged DNA during colorectal carcinogenesis. Generally speaking, factors of the same pathways possibly showed similar expression profiles. But XPF showed different preference (downregulation) with other NER factors (upregulation) according to ONCOMINE. The reason of this phenomenon might be that any mRNA level changes may not indicate the protein levels in a specific setting and that XPF might possess other functions out of NER pathways. Various posttranscriptional regulation including miRNA, lncRNA, and RNA methylation could affect the protein levels of a certain gene [25]. For example, miR-192 has been reported to inhibit nucleotide excision repair by targeting XPF in HepG2.2.15 cells [26]. Therefore, different expression profiles of XPF and other NER factors such as XPG found in ONCOMINE database might come from multiple posttranscriptional regulation. Whether certain changes of NER factor mRNA expression reflect corresponding protein levels still needs future studies to confirm. In addition, the phenomenon that XPF was downregulated in CRC tissues requires further large-scale studies to elucidate.

The relationship of ERCC2, ERCC3, ERCC5, XPA, and DDB2 with clinicopathological parameters of CRC we found in this study revealed the implication of NER members in the progression of CRC. Subjects with high ERCC2 expression were less likely to be observed in T3/T4 stage than low ERCC2 expression individuals in rectal cancer. For colon cancer, high ERCC3 expression was related to better T stage. Increased ERCC5 expression demonstrated significant predominance in worse T stage and presence of distant metastasis in colon cancer. In rectal cancer, high XPA expression predicted worse TNM stage, T stage, and N stage. Although XPA did not present tumor-normal differential expression in TCGA data base, one study has showed that XPA mRNA level was downregulated in 52 patients with Dukes' C colorectal cancer than matched normal tissues by TaqMan real-time quantitative PCR [27]. In colon cancer, DDB2 high expression indicated earlier TNM stage and absence of lymph node or distant metastasis, which was consistent with one cellular research that DDB2 decreased invasion of cancer mainly through inhibiting epithelial-mesenchymal transition (EMT) of colon cells [28].

ERCC2, ERCC4, and XPC expressions might predict prognosis of CRC according to our analysis on TCGA data. ERCC2 expression was associated with worse OS of colon cancer and subgroup analysis suggested a more significant result in males with a HR value of 1.84. In comparison to a large number of researches concerning ERCC2 polymorphisms in CRC [13, 29–31], only two studies explored whether ERCC2 expression correlated with survival of CRC patients after receiving chemotherapy. Huang et al. performed immunohistochemical staining of ERCC2 protein in 180 CRC patients but failed to construct relationship between ERCC2 and clinical outcome of CRC [32]. Another study carried out in 80 Egypt CRC patients detected both the mRNA and protein expressions of ERCC2 but found no significant relation between ERCC2 levels and OS or EFS (event free survival) [33]. In colon cancer, high expression of ERCC4 was associated with significantly favourable OS than those with low ERCC4 expression in males. As the distortion-recognizing factor, XPC complex recognizes DNA damage through sensing the DNA distortion. In this study, subjects with high XPC expression level suffer significantly decreased hazards of death for rectal cancer. 

These findings altogether suggested that aberrant changes of key factors involved in each step including recognition, unwinding, and excision of NER pathway demonstrate significant influence on CRC development and clinical outcome. As key genes involved in “recognition” step, XPA and DDB2 showed obvious relationship with TNM stage, while XPC expression indicated longer survival. The “unwinding” of damaged DNA is accomplished by ERCC2 and ERCC3, both of which negatively correlated with invasion depth of T stage. In addition, ERCC2 overexpression predicted worse prognosis. As for the NER members responsible for “excision” step, ERCC1 was overexpressed in CRC tissues. Colon cancer male patients with high ERCC4 expression showed favourable survival. Increased ERCC5 expression exhibited significant predominance in worse T stage and presence of distant metastasis. Recently, additional functions of NER factors outside the canonical NER pathway were identified. Chatzinikolaou et al. indicated that ERCC1-XPF cooperates with CTCF and cohesion to facilitate the developmental silencing of imprinted genes and that persistent DNA damage triggers chromatin changes that affect gene expression programs associated with NER disorders [34]. In addition, Kamileri et al. suggested that ERCC1-XPF is recruited on the promoters of genes associated with growth and ERCC1-XPF facilitates transcription initiation in vitro [35]. These findings provide novel implications of NER factors in cancer development and might help us understand the final outcome in CRC progression. Future molecular experiments concerning the biological functions of these key NER members in colorectal carcinogenesis and progression might generate promising significance.

In summary, core members of NER pathway might serve as novel biomarkers to indicate colorectal carcinogenesis and prognosis. Through comprehensive analysis of expression data from ONCOMINE and TCGA, we found that ERCC1, ERCC2, ERCC4, ERCC5, and DDB2 were differently expressed in CRC and normal tissues; ERCC2, ERCC3, ERCC5, XPA, and DDB2 correlated with clinicopathological parameters of CRC, while ERCC2, ERCC4, and XPC might predict prognosis of CRC. Future well-designed studies with large samples are still required to shed light on the significance of NER pathway members in CRC development and treatment.

## Figures and Tables

**Table 1:** Basic characteristics and function of key NER pathway genes.

Gene	Location	Exon	Protein mass	Step	Biological function
ERCC1	19q13.32	14	33 kDa	Incision	Incision of damaged DNA at 5′ site by forming XPF-ERCC1 heterodimer
ERCC2 (XPD)	19q13.32	24	86 kDa	Unwinding	5′-3′ unwinding of the DNA strands of the damaged site
ERCC3 (XPB)	2q14.3	15	89 kDa	Unwinding	3′-5′ unwinding of the DNA strands of the damaged site
ERCC4 (XPF)	16p13.12	13	104 kDa	Incision	Incision of damaged DNA at 5′ site by forming XPF-ERCC1 heterodimer
ERCC5 (XPG)	13q33.1	15	133 kDa	Incision	Incision of damaged DNA at 3′ site
ERCC6 (CSB)	10q11.23	23	168 kDa	Recognition	Initiation of TCNER, promote complex formation at DNA repair sites
ERCC8 (CSA)	5q12.1	13	44 kDa	Recognition	Initiation of TCNER, interacts with ERCC6
XPA	9q22.33	10	31 kDa	Recognition	Initiates repair by binding to DNA damaged sites
XPC	3p25.1	18	106 kDa	Recognition	Specifically participates in GGNER, XPC complex preferentially binds damaged DNA
DDB1	11q12.2	27	127 kDa	Recognition	Recognize UV-induced DNA damage by forming a complex with DDB2
DDB2	11p11.2	10	48 kDa	Recognition	Recognize UV-induced DNA damage by forming a complex with DDB1

**Table 2:** Significant changes of NER expression between different types of CRC and normal tissues.

Gene	Compared group	Up/down	Fold change	*t*-test	*P* value	Dataset
ERCC1	Colon adenocarcinoma versus normal	↑	3.075	11.744	1.67*E* − 13	TCGA colorectal
	Rectal adenocarcinoma versus normal	↑	3.813	12.954	1.79*E* − 16	TCGA
	Colon carcinoma versus normal	↑	2.391	8.249	1.68*E* − 06	Skrzypczak Colorectal 2

ERCC2	Colon adenoma versus normal	↑	2.813	8.805	3.18*E* − 12	Sabates-Bellver Colon
	Rectal adenoma versus normal	↑	3.008	7.979	7.63*E* − 08	Sabates-Bellver Colon

ERCC4	Rectosigmoid adenocarcinoma versus normal	↓	−2.271	−5.151	0.009	TCGA colorectal

ERCC5	Rectal mucinous adenocarcinoma versus normal	↑	2.121	4.362	0.005	Kaiser Colon

DDB2	Colon adenoma versus normal	↑	3.159	10.848	4.80*E* − 14	Sabates-Bellver Colon
	Rectal adenoma versus normal	↑	2.890	5.475	4.30*E* − 04	Sabates-Bellver Colon

**Table 3:** Association of NER pathway mRNA expression with clinicopathological parameters of CRC.

Cancer type	Gene	Expression	TNM			T			N			M		
			III-IV	I-II	*P*	T3/T4	TI/T2	*P*	Presence	Absence	*P*	Presence	Absence	*P*
Colon cancer	ERCC3	High	101	130		179	58		96	141		39	173	
		Low	97	137	0.621	202	36	**0.011 **	97	142	0.986	27	176	0.156
	ERCC5	High	107	121		199	38		106	132		41	160	
		Low	91	146	0.063	182	56	**0.040 **	87	151	0.076	25	189	**0.015**
	DDB2	High	85	150		190	47		84	154		24	186	
		Low	113	117	**0.005**	191	47	0.982	109	129	**0.020 **	42	163	**0.012**
Rectal cancer	ERCC2	High	37	42		58	25		38	44		13	65	
		Low	38	39	0.753	69	12	**0.019**	40	40	0.641	10	61	0.663
	XPA	High	44	33		69	12		47	33		9	63	
		Low	31	48	**0.025**	58	25	**0.019**	31	51	**0.008**	14	63	0.337

**Table 4:** Prognostic role of NER pathway mRNA expression in CRC.

Gene	Subgroup	Colon cancer				Rectal cancer			
		Univariate analysis		Multivariate analysis		Univariate analysis		Multivariate analysis	
		HR (95% CI)	*P*	Adjusted HR (95% CI)	*P*	HR (95% CI)	*P*	Adjusted HR (95% CI)	*P*
ERCC1		1.30 (0.88–1.93)	0.192	1.27 (0.84–1.90)	0.256	1.23 (0.57–2.65)	0.606	0.97 (0.40–2.34)	0.951
ERCC2		1.35 (0.91–2.00)	0.142	**1.53 (1.01–2.31)**	**0.043**	1.26 (0.58–2.73)	0.559	2.48 (0.90–6.82)	0.078
	Male			**1.84 (1.04–3.26)**	**0.037**				
	Female			1.22 (0.66–2.24)	0.532				
ERCC3		1.16 (0.79–1.71)	0.459	0.89 (0.60–1.34)	0.587	1.07 (0.49–2.35)	0.870	1.49 (0.59–3.77)	0.404
ERCC4		0.76 (0.51–1.12)	0.166	0.74 (0.49–1.11)	0.148	0.52 (0.23–1.14)	0.101	0.70 (0.28–1.76)	0.452
	Male			**0.54 (0.30–0.96)**	**0.035**				
	Female			1.15 (0.63–2.09)	0.654				
ERCC5		1.16 (0.79–1.72)	0.449	0.95 (0.63–1.42)	0.784	0.82 (0.38–1.78)	0.613	0.64 (0.26–1.56)	0.328
ERCC6		1.11 (0.76–1.64)	0.592	1.24 (0.83–1.84)	0.302	1.38 (0.63–3.02)	0.415	1.16 (0.48–2.80)	0.745
ERCC8		0.95 (0.64–1.39)	0.780	1.01 (0.67–1.52)	0.964	0.95 (0.44–2.07)	0.905	1.75 (0.71–4.34)	0.224
XPA		1.19 (0.80–1.77)	0.398	1.13 (0.75–1.71)	0.552	1.24 (0.56–2.74)	0.591	0.98 (0.36–2.68)	0.971
XPC		0.85 (0.58–1.25)	0.407	0.75 (0.50–1.12)	0.159	**0.40 (0.18–0.89)**	**0.026 **	0.55 (0.21–1.42)	0.214
	Male					**0.26 (0.08–0.88)**	**0.030 **		
	Female					0.61 (0.20–1.87)	0.385		
DDB1		0.73 (0.50–1.08)	0.118	0.87 (0.58–1.30)	0.488	0.62 (0.28–1.39)	0.242	0.90 (0.37–2.19)	0.811
DDB2		1.03 (0.70–1.52)	0.882	1.27 (0.84–1.92)	0.262	1.32 (0.61–2.86)	0.482	1.24 (0.51–2.99)	0.636

## Supplementary Materials

Supplementary Materials Supplementary Table  1. Association of NER pathway mRNA expression with clinicopathological parameters of colon cancer. Supplementary Table  2. Association of NER pathway mRNA expression with clinicopathological parameters of rectal cancer.
