# Supplementary material for: The Differential Expression of Core Genes in Nucleotide Excision Repair Pathway Indicates Colorectal Carcinogenesis and Prognosis
Source: Biomed Res Int. 2018 Jan 15;2018:9651320. doi: 10.1155/2018/9651320 (PMC5820669; doi:10.1155/2018/9651320)
Supplement: Supplementary Materials — Supplementary Table 1. Association of NER pathway mRNA expression with clinicopathological parameters of colon cancer. Supplementary Table 2. Association of NER pathway mRNA expression with clinicopathological parameters of rectal cancer. [file 9651320.f1.docx]

Supplementary Table 1. Association of NER pathway mRNA expression with clinicopathological parameters of colon cancer.

|  |  | **TNM** | | | **T** | | | **N** | | | **M** | | | **Recurrence** | | |
| --- | --- | --- | --- | --- | --- | --- | --- | --- | --- | --- | --- | --- | --- | --- | --- | --- |
| **Gene** | **Expression** | **III-IV** | **I-II** | **P** | **T3/T4** | **TI/T2** | **P** | **Presence** | **Absence** | **P** | **Presence** | **Absence** | **P** | **Presence** | **Absence** | **P** |
| ERCC1 | High | 99 | 134 |  | 194 | 44 |  | 96 | 142 |  | 33 | 170 |  | 10 | 228 |  |
|  | Low | 99 | 133 | 0.968 | 187 | 50 | 0.475 | 97 | 141 | 0.926 | 33 | 179 | 0.848 | 10 | 228 | 1.000 |
| ERCC2 | High | 98 | 134 |  | 193 | 44 |  | 93 | 144 |  | 33 | 171 |  | 12 | 225 |  |
|  | Low | 100 | 133 | 0.883 | 188 | 50 | 0.504 | 100 | 139 | 0.563 | 33 | 178 | 0.881 | 8 | 231 | 0.351 |
| ERCC3 | High | 101 | 130 |  | 179 | 58 |  | 96 | 141 |  | 39 | 173 |  | 12 | 225 |  |
|  | Low | 97 | 137 | 0.621 | 202 | 36 | **0.011** | 97 | 142 | 0.986 | 27 | 176 | 0.156 | 8 | 231 | 0.351 |
| ERCC4 | High | 92 | 141 |  | 186 | 52 |  | 89 | 150 |  | 32 | 176 |  | 8 | 231 |  |
|  | Low | 106 | 126 | 0.176 | 195 | 42 | 0.259 | 104 | 133 | 0.140 | 34 | 173 | 0.772 | 12 | 225 | 0.351 |
| ERCC5 | High | 107 | 121 |  | 199 | 38 |  | 106 | 132 |  | 41 | 160 |  | 13 | 225 |  |
|  | Low | 91 | 146 | 0.063 | 182 | 56 | **0.040** | 87 | 151 | 0.076 | 25 | 189 | **0.015** | 7 | 231 | 0.170 |
| ERCC6 | High | 90 | 142 |  | 193 | 44 |  | 87 | 151 |  | 31 | 167 |  | 11 | 227 |  |
|  | Low | 108 | 125 | 0.099 | 188 | 50 | 0.504 | 106 | 132 | 0.076 | 35 | 182 | 0.895 | 9 | 229 | 0.648 |
| ERCC8 | High | 98 | 136 |  | 191 | 46 |  | 94 | 143 |  | 38 | 175 |  | 8 | 229 |  |
|  | Low | 100 | 131 | 0.759 | 190 | 48 | 0.836 | 99 | 140 | 0.696 | 28 | 174 | 0.268 | 12 | 227 | 0.371 |
| XPA | High | 91 | 141 |  | 194 | 44 |  | 89 | 149 |  | 34 | 171 |  | 11 | 227 |  |
|  | Low | 107 | 126 | 0.144 | 187 | 50 | 0.475 | 104 | 134 | 0.161 | 32 | 178 | 0.708 | 9 | 229 | 0.648 |
| XPC | High | 92 | 138 |  | 190 | 48 |  | 92 | 147 |  | 27 | 170 |  | 11 | 228 |  |
|  | Low | 106 | 129 | 0.266 | 191 | 46 | 0.836 | 101 | 136 | 0.360 | 39 | 179 | 0.244 | 9 | 228 | 0.662 |
| DDB1 | High | 100 | 133 |  | 184 | 54 |  | 94 | 144 |  | 30 | 174 |  | 11 | 227 |  |
|  | Low | 98 | 134 | 0.883 | 197 | 40 | 0.112 | 99 | 139 | 0.641 | 36 | 175 | 0.512 | 9 | 229 | 0.648 |
| DDB2 | High | 85 | 150 |  | 190 | 47 |  | 84 | 154 |  | 24 | 186 |  | 8 | 230 |  |
|  | Low | 113 | 117 | **0.005** | 191 | 47 | 0.982 | 109 | 129 | **0.020** | 42 | 163 | **0.012** | 12 | 226 | 0.361 |

Supplementary Table 2. Association of NER pathway mRNA expression with clinicopathological parameters of rectal cancer.

|  |  | **TNM** | | | **T** | | | **N** | | | **M** | | | **Recurrence** | | |
| --- | --- | --- | --- | --- | --- | --- | --- | --- | --- | --- | --- | --- | --- | --- | --- | --- |
| **Gene** | **Exression** | **III-IV** | **I-II** | **P** | **T3/T4** | **TI/T2** | **P** | **Presence** | **Absence** | **P** | **Presence** | **Absence** | **P** | **Presence** | **Absence** | **P** |
| ERCC1 | High | 34 | 44 |  | 59 | 23 |  | 37 | 44 |  | 14 | 62 |  | 4 | 79 |  |
|  | Low | 41 | 37 | 0.262 | 68 | 14 | 0.093 | 41 | 40 | 0.529 | 9 | 64 | 0.304 | 3 | 79 | 0.711 |
| ERCC2 | High | 37 | 42 |  | 58 | 25 |  | 38 | 44 |  | 13 | 65 |  | 4 | 79 |  |
|  | Low | 38 | 39 | 0.753 | 69 | 12 | **0.019** | 40 | 40 | 0.641 | 10 | 61 | 0.663 | 3 | 79 | 0.711 |
| ERCC3 | High | 39 | 44 |  | 63 | 20 |  | 38 | 45 |  | 15 | 65 |  | 3 | 80 |  |
|  | Low | 36 | 37 | 0.772 | 64 | 17 | 0.634 | 40 | 39 | 0.537 | 8 | 61 | 0.228 | 4 | 78 | 0.687 |
| ERCC4 | High | 38 | 39 |  | 64 | 19 |  | 40 | 41 |  | 12 | 61 |  | 4 | 79 |  |
|  | Low | 37 | 42 | 0.753 | 63 | 18 | 0.918 | 38 | 43 | 0.753 | 11 | 65 | 0.740 | 3 | 79 | 0.711 |
| ERCC5 | High | 41 | 35 |  | 63 | 18 |  | 44 | 36 |  | 10 | 60 |  | 6 | 76 |  |
|  | Low | 34 | 46 | 0.153 | 64 | 19 | 0.918 | 34 | 48 | 0.085 | 13 | 66 | 0.714 | 1 | 82 | **0.051** |
| ERCC6 | High | 35 | 41 |  | 65 | 16 |  | 37 | 43 |  | 13 | 57 |  | 6 | 76 |  |
|  | Low | 40 | 40 | 0.622 | 62 | 21 | 0.395 | 41 | 41 | 0.633 | 10 | 69 | 0.319 | 1 | 82 | **0.051** |
| ERCC8 | High | 36 | 41 |  | 61 | 21 |  | 37 | 43 |  | 10 | 65 |  | 2 | 81 |  |
|  | Low | 39 | 40 | 0.744 | 66 | 16 | 0.35 | 41 | 41 | 0.633 | 13 | 61 | 0.474 | 5 | 77 | 0.240 |
| XPA | High | 44 | 33 |  | 69 | 12 |  | 47 | 33 |  | 9 | 63 |  | 3 | 79 |  |
|  | Low | 31 | 48 | **0.025** | 58 | 25 | **0.019** | 31 | 51 | **0.008** | 14 | 63 | 0.337 | 4 | 79 | 0.711 |
| XPC | High | 37 | 41 |  | 63 | 19 |  | 38 | 43 |  | 8 | 65 |  | 6 | 77 |  |
|  | Low | 38 | 40 | 0.873 | 64 | 18 | 0.852 | 40 | 41 | 0.753 | 15 | 61 | 0.138 | 1 | 81 | 0.056 |
| DDB1 | High | 40 | 41 |  | 66 | 17 |  | 40 | 42 |  | 15 | 64 |  | 2 | 81 |  |
|  | Low | 35 | 40 | 0.734 | 61 | 20 | 0.519 | 38 | 42 | 0.87 | 8 | 62 | 0.202 | 5 | 77 | 0.240 |
| DDB2 | High | 34 | 44 |  | 59 | 22 |  | 36 | 45 |  | 10 | 65 |  | 1 | 81 |  |
|  | Low | 41 | 37 | 0.262 | 68 | 15 | 0.164 | 42 | 39 | 0.345 | 13 | 61 | 0.474 | 6 | 77 | 0.056 |
